# Coffee as a Stimulant

**Published:** 1885-02

**Authors:** 


### COFFEE AS A STIMULANT.

The cup of coffee, provided it were genuine, contained more alkaloidal stimulant than the cup of tea, and owing to the absence o tannin the action of coffee was more rapid than that of tea. The specific gravity of a cup of tea was about 1.003, that of strong coffee 1.009, and of *cafe au lait*, sweetened, 1.035. Tea was more of a pure beverage than coffee, and hence it was possible to use it as a mere luxury, for it required scarcely any digestive effort, and did not "cloy" the palate. The danger of excessive tea drinking lay mainly in the large amount of astringent matter. This was a most potent cause of dyspepsia among women of the seamstress class, who frequently consumed tea which had been boiled. When the system stood in need of a stimulant there was nothing equal to a cup of strong coffee; and if it were desired to wean the drunkard from his spirits a real stimulant must be supplied, and not the sickly, bitter, unwholesome stuff which was called "coffee" in this country. In order to make good coffee the berry must be fresh roasted and ground. There was no difficulty whatever in roasting coffee and this ought to be part of the daily routine of every well-regulated household. It was important to use enough coffee; one and a half to two ounces of coffee to a pint of water made a first-rate beverage. Elaborate coffee machines for grinding were by no means necessary. If the coffee required for breakfast were put into a common earthenware jug over night and cold water poured upon it, it might be heated to the boiling point in the morning by being allowed to stand in a saucepan of water over the fire. Violent ebullition was thus avoided, and the aroma was preserved. Chicory and other allied bodies are in no way substitutes for coffee, for they possess no stimulant properties. Out of ninety samples of ground coffee purchased in London shops only five were found to be genuine.—*The Lancet.*

